# Investigating the conformational landscape of AlphaFold2-predicted protein kinase structures

**DOI:** 10.1093/bioadv/vbad129

**Published:** 2023-09-15

**Authors:** Carmen Al-Masri, Francesco Trozzi, Shu-Hang Lin, Oanh Tran, Navriti Sahni, Marcel Patek, Anna Cichonska, Balaguru Ravikumar, Rayees Rahman

**Affiliations:** Harmonic Discovery Inc., New York, NY 10013, United States; Department of Physics and Astronomy, University of California Irvine, Irvine, CA 92697, United States; Harmonic Discovery Inc., New York, NY 10013, United States; Harmonic Discovery Inc., New York, NY 10013, United States; Department of Chemical Engineering, University of Michigan Ann Arbor, Ann Arbor, MI 48109, United States; Harmonic Discovery Inc., New York, NY 10013, United States; Department of Chemistry, University of California Irvine, Irvine, CA 92697, United States; Harmonic Discovery Inc., New York, NY 10013, United States; Harmonic Discovery Inc., New York, NY 10013, United States; Harmonic Discovery Inc., New York, NY 10013, United States; Harmonic Discovery Inc., New York, NY 10013, United States; Harmonic Discovery Inc., New York, NY 10013, United States

## Abstract

**Summary:**

Protein kinases are a family of signaling proteins, crucial for maintaining cellular homeostasis. When dysregulated, kinases drive the pathogenesis of several diseases, and are thus one of the largest target categories for drug discovery. Kinase activity is tightly controlled by switching through several active and inactive conformations in their catalytic domain. Kinase inhibitors have been designed to engage kinases in specific conformational states, where each conformation presents a unique physico-chemical environment for therapeutic intervention. Thus, modeling kinases across conformations can enable the design of novel and optimally selective kinase drugs. Due to the recent success of AlphaFold2 in accurately predicting the 3D structure of proteins based on sequence, we investigated the conformational landscape of protein kinases as modeled by AlphaFold2. We observed that AlphaFold2 is able to model several kinase conformations across the kinome, however, certain conformations are only observed in specific kinase families. Furthermore, we show that the per residue predicted local distance difference test can capture information describing structural flexibility of kinases. Finally, we evaluated the docking performance of AlphaFold2 kinase structures for enriching known ligands. Taken together, we see an opportunity to leverage AlphaFold2 models for structure-based drug discovery against kinases across several pharmacologically relevant conformational states.

**Availability and implementation:**

All code used in the analysis is freely available at https://github.com/Harmonic-Discovery/AF2-kinase-conformational-landscape.

## 1 Introduction

Protein kinases are a family of more than 500 proteins that catalyze the transfer of a phosphate group to their substrates, switching specific cellular pathways on or off. As a result of mutations, differential expression or other forms of dysregulation, kinases are known to cause diseases, such as cancer and autoimmunity (Ferguson and Gray 2018, Zarrin *et al.* 2021). Consequently, they are one of the largest drug target families in the druggable genome (Cohen 2002). Importantly, kinases are structurally dynamic proteins that can adopt several conserved active and inactive conformational states. These specific conformations regulate important aspects of cellular physiology and are key driving factors for protein–protein (Röck *et al.* 2019) and protein–ligand interactions (Haldane *et al.* 2016).

In the active conformation of protein kinases, the conserved DFG motif and αC helix have an “in” conformation (CIDI). This means that the conserved phenylalanine of the DFG motif (DFG-Phe) points out of the active site while the aspartate (DFG-Asp) faces the ATP-binding site. Additionally, the conserved glutamic acid in the center of αC helix (C-Glu) forms a salt bridge with the conserved β3-lysine. Conversely, inactive conformations comprise either the DFG or αC helix adopting the “out” conformation (CIDO, CODI, and CODO) (Ung *et al.* 2018). That is, the directionality of the DFG-Asp and DFG-Phe are flipped, or the glutamic acid in the αC helix breaks contact with β3-lysine. Additionally, an inactive conformation can arise when the DFG-Phe adopts an intermediate conformation (ωCD) (Ung *et al.* 2018).

Inhibitors binding to these distinct conformational states are also well-characterized. For example, type I inhibitors are ATP competitive molecules that bind to the active CIDI conformation; whereas type I$\frac{1}{2}$ and type II inhibitors engage the inactive CODI or CIDO conformations, respectively (Roskoski 2016). These distinct inhibitor types occupy diverse regions of the chemical space and confer pharmacokinetic or dynamic advantages (Roskoski 2016). Therefore, approaches that accurately model the different conformational states of kinases can enable the rational design of novel, conformation-specific inhibitors.

Of the 3700 crystal structures of human protein kinases in the Protein Data Bank (PDB) (Berman *et al.* 2000), roughly half of the human kinome is covered (Ung *et al.* 2018). Additionally, over 60% of crystal structures catalogue kinases in just their active conformation (Modi and Dunbrack 2019a). Due to the striking success of AlphaFold2 (AF2) in the prediction of protein structure from sequence (Jumper *et al.* 2021), and AF2’s accuracy in modeling membrane transporter conformational diversity (Del Alamo *et al.* 2022), we hypothesized that AF2 may be able to model the conformational landscape of protein kinases. In this work, we performed a conformational analysis of all the modeled protein kinase structures from the AlphaFold Protein Structure Database (AlphaFoldDB) (Varadi *et al.* 2022). Furthermore, we investigated the link between the predicted local distance difference test (pLDDT), an AF2 confidence metric, and the intrinsic flexibility of residues in kinases. We then evaluated their utility in structure-based drug discovery (SBDD) by calculating the enrichment of known actives to selected kinases using molecular docking. The enrichment observed in the AF2 structures was then compared to the best matched native crystal structure from the PDB.

## 2 Methods

### 2.1 Acquisition and prediction of conformation of PDB and AF2 protein kinase structures

All protein kinase structures present in both the PDB (Berman *et al.* 2000) and AlphaFoldDB (Varadi *et al.* 2022) (https://alphafold.ebi.ac.uk/) were recovered. Using the SCOPe classification (Chandonia *et al.* 2019), only chains containing the “Protein kinases, catalytic subunit” family were retained from the PDB, and the catalytic domain was subsequently extracted from these chains. This filtering process resulted in 5752 unique PDB IDs from which 8460 chains were selected.

From AlphaFoldDB, only the structures containing a “Protein Kinase” domain annotated by UniProtKB (UniProt Consortium 2015) were retained. The catalytic domains of these structures were extracted. After this initial filtering step, 4348 structures remained. Kinformation (Ung *et al.* 2018, Rahman *et al.* 2019) was used to annotate the different conformations of kinase domains. Kinformation is a random forest model, which uses descriptors related to the αC helix and DFG motif to predict the conformation of kinases. Kinformation was set to discard sequences with a length <225 residues, and <40% sequence identity to all canonical human kinases. Structures that passed these filtering criteria were subsequently aligned with the Modi–Dunbrack alignment (Modi and Dunbrack 2019b) using MUSCLE (Edgar 2004), and the alignment was further refined through structural alignment using PyMOL (Schrödinger LLC 2015), with 1ATP as reference.

### 2.2 Binding pocket comparison of AF2-modeled kinase structures

The structures predicted by AF2 were clustered by binding pocket similarity. The binding pocket of each kinase was characterized by a set of 85 residues, as defined by KLIFS (Kooistra *et al.* 2016). These residues characterize the ligand–receptor interactions of type I, I$\frac{1}{2}$, II, III, and most type IV inhibitors. The 85-residue sequence for each kinase was aligned to their corresponding AF2 structure using Bio.pairwise2 (Cock *et al.* 2009) (global sequence alignment with a match score of 1, a mismatch penalty of −1, a gap penalty of −4, and no gap extension penalty).

After obtaining the binding pocket residues, we utilized the KiSSim package (Sydow *et al.* 2022a) to extract spatial properties of each pocket residue (default settings). The spatial properties consisted of the distances of each residue to the center of the pocket, to the hinge region, to the front pocket, and to the DFG region. The structures were clustered by using t-SNE (Van der Maaten and Hinton 2008) (5000 iterations, perplexity of 30, 3 components).

### 2.3 Molecular docking and crystal structure selection workflow

Docking enrichment calculations were carried out for three kinases: ABL1, BTK, and DDR1. For each kinase, a set of representative structures was selected as a result of the following steps: (i) all holo-structures were initially considered; (ii) structures with missing or mutated residues in the binding pocket or had a resolution >2.5 Å were excluded; (iii) each crystal structure was assigned a conformational state following the scheme proposed by Ung *et al.* (2018), and featurized using fpocket (Peter *et al.* 2009) along with KLIFS descriptors for the αC helix, DFG, and Glycine rich regions (OpenCADD-KLIFS) (Sydow *et al.* 2022b); (iv) these structures were clustered using DBSCAN (Ester *et al.* 1996). The representative structure for a given conformation cluster was determined to be the structure that maximized the total number of protein–ligand interactions; and (v) finally, the representative structures were subjected to energy minimization using the Molecular Operating Environment (Chemical Computing Group 2022).

These structures were evaluated against benchmark sets of known actives, inactives, and decoys built for each kinase target. A subset of active molecules with known activity <1000 nM (IC_50_, K${}_{i}$, or K${}_{d}$) were selected using a diversity picker implemented in RDKit (Landrum *et al.* 2013) (20% of known actives with <0.20 average Tanimoto similarity). All known inactives with activity >9999 nM were selected, and decoys were generated using DeepCoy (Imrie *et al.* 2021) pre-trained on the DUDE model to achieve a total of 25 negative examples per active. The benchmark set was neutralized using RDKit, and the 3D conformers were generated using Open Babel (O’Boyle *et al.* 2011).

The docking calculations were performed using Smina (Koes *et al.* 2013). Prior to docking, all representative protein structures were superimposed, and all orthosteric ligands were considered to define the consensus binding site. Smina default scoring function and parameters were employed. For each kinase, an early enrichment analysis was performed on structures belonging to the same conformational state of the AF2 structure. The structures with the best enrichment were then selected to be compared to the AF2-generated ones. To calculate the docking enrichment curves, the docked ligands were ranked based on their binding affinity. The ratio of known actives against the total number of compounds was plotted to test the capability of each structure to discriminate actives from inactives and decoys.

Lastly, the ability of AF2 structures to reproduce pharamacologically relevant interactions was assessed via cross-docking analysis on a set of 30 kinases. To evaluate crystal structures, each crystal ligand was docked to all crystal structure of the same kinase, except for its native structure. For each AF2 structure, all crystal ligands of the same kinase were docked to it. The reproduction of interactions was assessed by computing protein–ligand interaction fingerprints using the protein–ligand interaction profiler package (Salentin *et al.* 2015) and compared to native ligand contacts using a Jaccard similarity score.

### 2.4 Molecular Dynamics simulations

The AF2 structure of ABL1 from the AlphaFoldDB (Varadi *et al.* 2022) served as the initial structure for our Molecular Dynamics (MD) simulations. The terminal regions of the structure were truncated to retain only the kinase catalytic domain and capped using ACE and NME for the N-terminal and C-terminal, respectively. Two systems were created: an apo system and a holo system, where the crystallized ligand from the PDB structure 2F4J was placed in the binding pocket.

To prepare the ligand for simulation, we used the antechamber package in AMBER (Case *et al.* 2022), employing the AM1-BCC method to assign partial atomic charges and the General Amber Force Field 2 to assign force field parameters. The protein was parameterized using the Amber ff19SB force field. Titratable residues’ protonation states were determined using the H++ web tool (http://newbiophysics.cs.vt.edu/H++/) (Anandakrishnan *et al.* 2012). The systems were solvated with OPC water molecules, ensuring a minimum distance of 12 (Å) in each axis from the closest protein atom. The system’s charge was neutralized using sodium cations. To reproduce a salt concentration of 0.15 M, chloride anions and sodium cations were added.

Prior to the production phase, we conducted a stepwise minimization of the solvent and salt using the steep descent method for 20 cycles, followed by the conjugate gradient minimization for a maximum of 1000 cycles. Subsequently, the entire system underwent minimization using the same procedure. We initiated a 50-ps canonical ensemble (NVT) MD simulation to raise the temperature from 0 to 300 K, which was maintained for the subsequent steps. The NVT simulations were carried out using the Langevin scheme with a collision frequency of 1.

For each structure, 100 ps isothermal–isobaric ensemble (NPT) equilibration dynamics were performed to stabilize the system density at 1 bar of pressure, using the Berendsen barostat with a pressure relaxation time of 2 ps; 100 ns of production phase in the isothermal–isobaric ensemble (NPT) was initiated using the final coordinates and velocities from the equilibration phase.

All simulations were performed using Amber22, with minimization and equilibration conducted using the sander module on the CPU. For production simulations, we employed the GPU CUDA-accelerated pmemd module. Throughout the simulations, we applied the SHAKE constraint for hydrogen covalent bonds with a time step of 2 fs. A cutoff of 10 Å was used for the calculation of non-bonded interactions.

Root mean squared deviation (RMSD) and root mean squared fluctuation (RMSF) were performed using MDAnalysis (Michaud-Agrawal *et al.* 2011). The interaction analysis between the protein structure and the ligand was performed using ProLIF (Bouysset and Fiorucci 2021). Structural renderings were done using 3D protein imaging (Tomasello *et al.* 2020).

## 3 Results

### 3.1 The conformational landscape of protein kinase models generated by AF2

**Figure 1.**
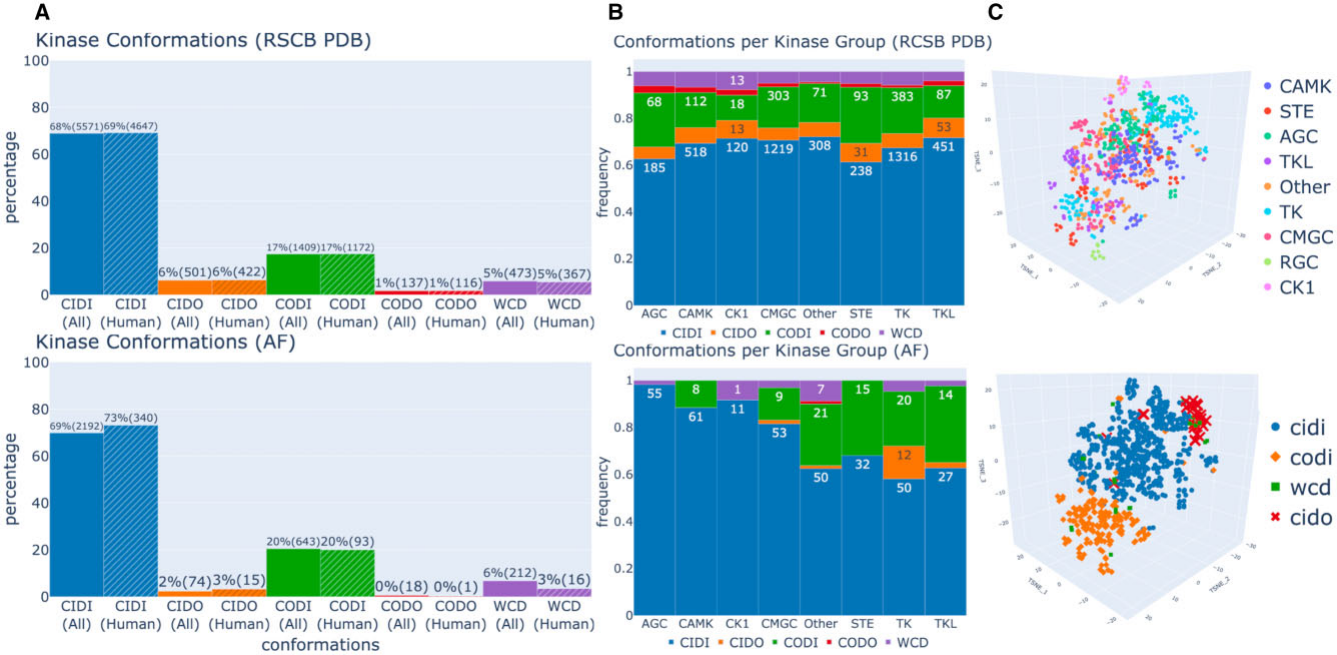
The conformational landscape of protein kinases. (A) The conformational diversity of kinase structures in the RCSB PDB database (top) and AlphaFoldDB (bottom) across all species (non-dashed bar) and Human (*Homo Sapiens*) species (dashed bar). (B) The proportion of each conformation across kinase families in the human kinome. (C) Projection and clustering of all human and mouse kinase structures using t-SNE, colored by kinase family (top) or conformation (bottom).

To investigate the conformational landscape of protein kinases, we predicted the different conformational states across both human and non-human protein kinase structures present in the RSCB PDB and the AlphaFoldDB databases. We observe that the conformational diversity of AF2-modeled protein kinase structures closely mirrored the proportion of kinase conformations in the PDB (Fig. 1A). Here, the active kinase conformation, CIDI, is highly over-represented in both AF2-modeled structures and the PDB (69% and 68%, respectively), followed by the CODI conformation (20% and 17%). Interestingly, CIDO structures are under-represented in AF2 compared to the PDB structures. This observation is also reflected in related work by Modi and Dunbrack, where they observe a large proportion of AF2 models of the human protein kinome are in the DFG-in active conformation (70.8%) while inactive DFG-out structures are under-represented (Modi and Dunbrack 2022).

The representation of kinase conformation is also dependent on the protein kinase family, where certain kinase families enrich for specific conformational states (Fig. 1B). In the PDB, we observe a relatively consistent proportion of CIDO (type II) kinase structures across all kinase families, as well as other rarer conformations, such as CODO and ωCD. However, cross-family conformational diversity is not necessarily conserved in the AF2 models. Specifically, CIDO and CODO conformations are not observed in AGC, CAMK, CK1, and STE families. However, in TK, the relative proportion of CIDO structures is higher in the AF2 models than the PDB structures. We hypothesize that this may be due to a larger diversity of ligands available for TK family kinases that stabilize the CIDO conformation.

We also investigated the differences in binding pockets across all human and mouse kinases in AF2 models by considering the distances of each of the residues belonging to the pocket to the center, hinge, DFG, and front pocket regions of the kinase (Fig. 1C). After projecting this high-dimensional feature space to three dimensions using t-SNE, we found that kinases belonging to the same family form clear sub-clusters (Fig. 1C, top). Many of the kinase families form 2–3 sub-clusters, most notably TK, TKL, and CAMK. Only the “Other” family does not cluster together and is spread across the t-SNE projected conformational space. We also find that these observed sub-clusters can be attributed to different conformations of kinases within the same family (CIDI, CODI, and CIDO) (Fig. 1C, bottom), with the structures in the intermediate conformation ωCD spread across the t-SNE space.

### 3.2 pLDDT as a measure of conformational plasticity in the protein kinase active site

Each residue of any AF2-modeled structure has an associated estimate of the confidence for its predicted 3D positioning, the pLDDT. Previous work by Binder *et al.* has shown that pLDDT scores can correlate to structural properties, such as protein disorder (Binder *et al.* 2022). Hegedűs *et al.* (2022), Guo *et al.* (2022), and Saldaño *et al.* (2022) have also shown a relationship between pLDDT and protein conformational dynamics. Since AF2 can model several pharmacologically relevant kinase conformational states (Fig. 1), we hypothesized if pLDDT correlated with the conformational space sampled by the available crystal structures for each kinase. For example, ABL1 currently has 27 human and mouse structures in the DFG-out conformation, 9 structures in the DFG-in conformation, and 16 structures in an intermediate conformation.

**Figure 2.**
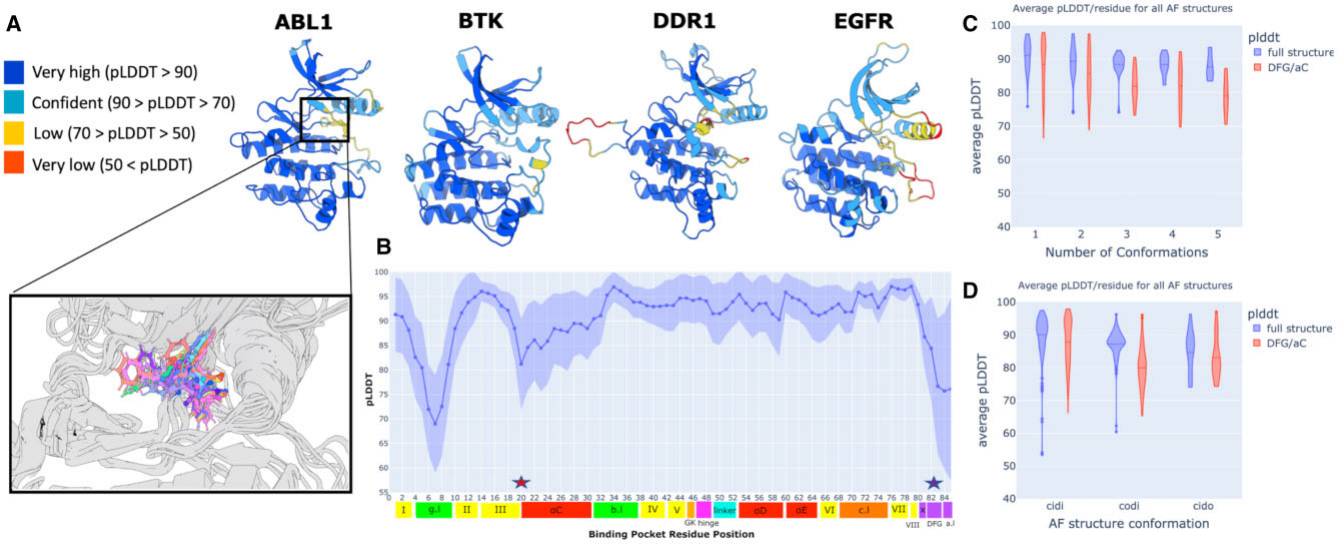
pLDDT as a measure of conformational plasticity. (A) pLDDT for all residues belonging to the protein kinase domain of ABL1, BTK, DDR1 and EGFR. The DFG motif is shown in licorice representation. The variation of the DFG-Phe residue across all human ABL1 structures is also shown (A, bottom). (B) The average pLDDT of the 85 binding pocket residues across all AF2-predicted structures. The standard deviation in the pLDDT is shown as the shaded region. The DFG-Phe (residue 20) and C-Glu (residue 83) motifs are marked by the red and purple stars, respectively. (C) Violin plot of the distribution of the average pLDDT across kinases, grouped by the number of conformations available for each kinase. (D) Violin plot of the distribution of the average pLDDT across kinases, grouped by the conformation of each structure. In both (C) and (D), the average pLDDT was computed across all the residues in the structure (blue) or across the residues part of the DFG motif and α-C helix (red).

We visualized AF2-modeled structures of four kinases: ABL1, BTK, DDR1, and EGFR. These kinases belong to different kinase families and are representative of multiple conformational states of the binding pocket. We observed variability of pLDDT scores in conformationally flexible regions of the pocket, specifically, in the relative positioning of the residues belonging to the DFG motif (residues 82–84) (Fig. 2A, bottom). We then evaluated the binding pocket pLDDT scores across all AF2 kinase structures and observed that DFG-Phe and C-Glu motifs (residues 20 and 83 in Fig. 2B) have higher uncertainty on average (∼75%) compared to the rest of the protein.

We proceeded to analyze if the variability of pLDDT scores in the binding pocket was either related to the number of unique conformations observed in the PDB for each kinase, or was a function of certain under-represented conformational states, such as CIDO. Figure 2C shows a clear relationship between the average pLDDT of either the whole kinase domain and DFG/αC helix motifs to the number of unique conformations observed in the PDB of a given kinase structure (Fig. 2C). As more conformations related to the movements of either the DFG or the αC helix are observed, per kinase, there is a drop in pLDDT at these residues for AF2 modeled structures. Likewise, under-represented kinase conformations on average have lower pLDDT in both their whole kinase domain structure and just their DFG/αC helix motifs (Fig. 2D).

Moreover, the relationship between conformational flexibility and pLDDT is further substantiated through an examination of the correlation between pLDDT and the B-factors observed in high quality ABL1, BTK, and EGFR crystal structures (Supplementary Fig. S1). B-factors, in crystal structures, serve as indicators of protein flexibility, reflecting the electron density spread for each atom. Our analysis reveals an intriguing anti-correlation between B-factors and pLDDT values (Supplementary Fig. S1), suggesting that regions with low modeled confidence, as predicted by AF2, experience notable atomic displacement within the crystal packing, and *vice versa*.

### 3.3 MD simulations confirm the stability of ABL1 AF2 structure

**Figure 3.**
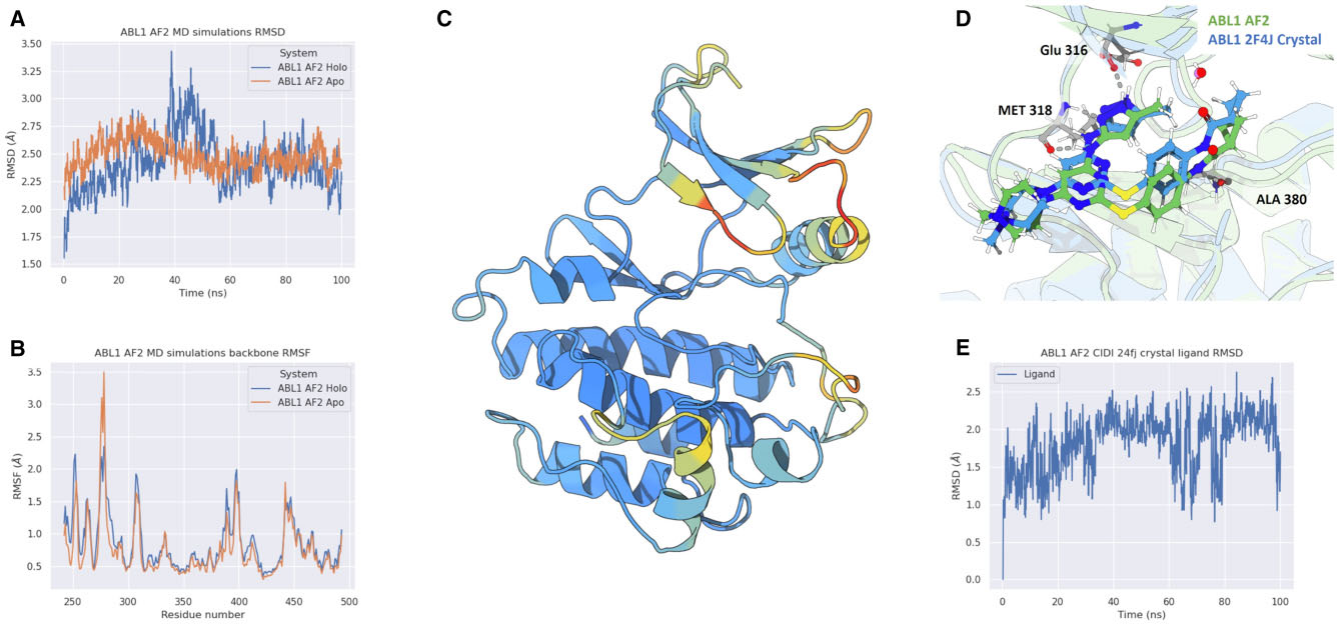
MD simulations of ABL1 CIDI AF2 structure in presence and absence of type I ligand. (A) RMSD of the protein atoms during the simulations. Blue line represents the simulation of the AF2 structure in presence of the type I ligand taken from the 2F4J PDB structure. The orange line represents the simulation of protein structure without ligand bound. (B) RMSF of the Cα atoms during the simulations. The blue line represents the simulation of the AF2 structure in presence of the type I ligand taken from the 2F4J PDB structure. Orange line represents the simulation of protein structure without ligand bound. (C) Structural visualization of the RMSF projected onto the ABL1 AF2 structure. Regions characterized by low fluctuations are colored in blue, while regions characterized by high fluctuations are colored in red. Light blue and yellow represent moderately rigid and moderately flexible regions, respectively. (D) Snapshot of the ligand in the 2F4J crystal structure (blue) and in a simulation frame (green). (E) RMSD of the 2F4J ligand within the AF2 binding site during the MD simulation.

It is crucial to acknowledge that crystal conditions might significantly differ from physiological conditions. To address this, MD simulations were employed to study the protein’s dynamics in a fully solvated environment and free from crystallographic constraints (as described in Section 2). In addition to the B-factors, our observations show that atomic fluctuations captured during MD simulations, analyzed through a root mean squared analysis (RMSF) of the backbone atoms, also exhibit an anti-correlation with pLDDT (Fig. 3B and C and Supplementary Fig. S2). It is worth noting that the DFG motif appears to be an exception, as it remains rigid over the course of the simulations. This discrepancy can be attributed to the need for more extended simulations or enhanced sampling techniques to capture the switching behavior of this particular structural motif.

During the simulation period, we noticed that the systems reached convergence with only minor deviations from the initial structures generated by AF2 (Fig. 3A). This finding suggests that the ABL1 structure produced by AF2 is already very close to an energy minimum. To assess the capability of the AF2 structure to accommodate a type I ligand, we conducted MD simulations of the ligand within the pocket of the AF2 structure. The process involved superimposing the AF2 structure onto the crystal ABL1 CIDI structure 2F4J, followed by ligand minimization and application of the simulation protocol detailed in Section 2. Throughout the simulations, we observed the ligand’s stability within the pocket, exhibiting minimal deviation from the starting structure (Fig. 3E). Analyzing the interactions (Fig. 3D and Supplementary Fig. S3), we found that the crucial interactions between the ligand and the protein, as observed in the crystal structure, were conserved in the AF2 structure during the simulation. The results of the simulations provide an indication that CIDI structures from AF2 can be leveraged for SBDD.

### 3.4 Comparison of ligand enrichment in selected, conformationally diverse AF2 kinase models against matched crystal structures

AF2 opens the door to conformation-specific SBDD. To evaluate if these predicted structures can be leveraged for virtual screening, we compared the docking enrichment of the predicted AF2 structures, to the highest scoring minimized crystal structure in terms of docking enrichment for three conformationally diverse kinases (ABL1, BTK, and DDR1).

**Figure 4.**
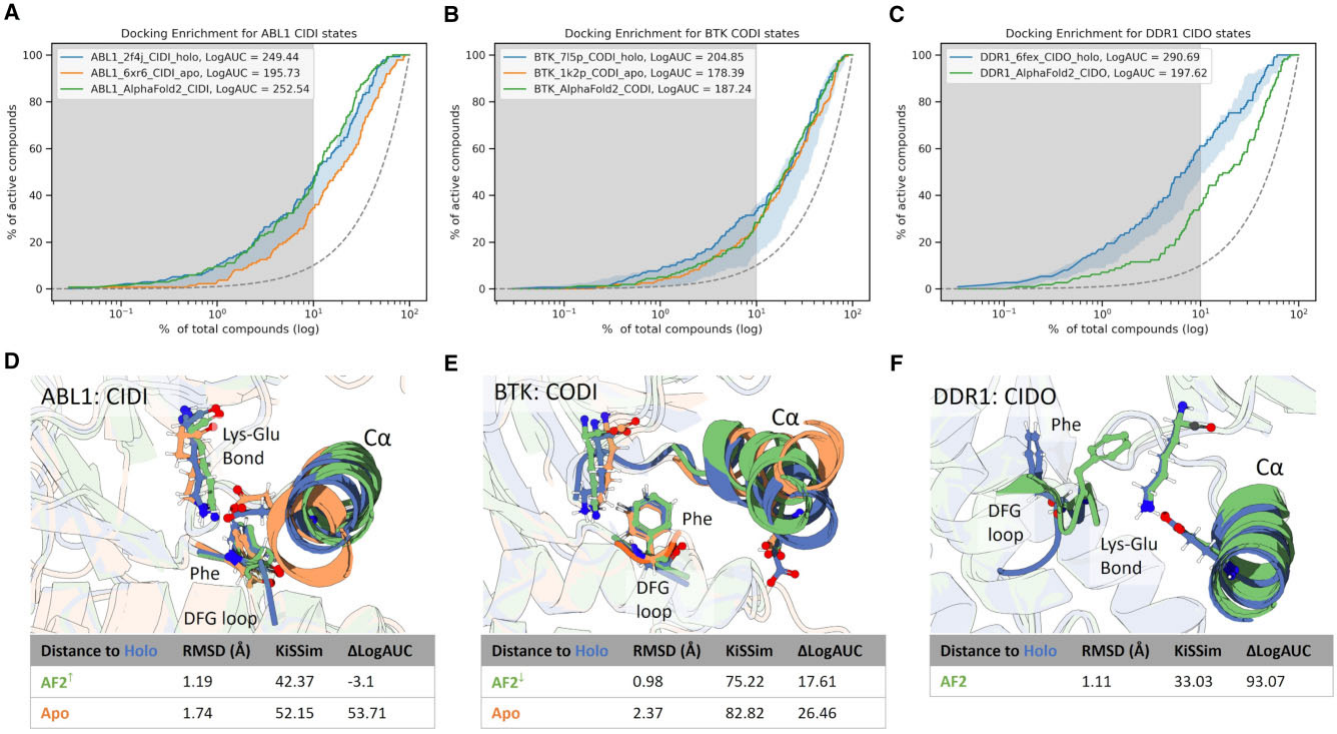
Docking enrichment curves for AF2-generated structures versus minimized crystal representatives. (A–C) Docking enrichment curves for ABL1 (CIDI), BTK (CODI), and DDR1 (CIDO). Blue lines represent enrichments for highest scoring structure, orange lines represent enrichments for apo structures, and green lines describe AF2 models. The blue shaded region represents the range of enrichment scores observed for all crystal structures. (D–F) Structural superimposition of the cα-Helix, DFG, and Lys-Glu hydrogen bond for ABL1 (CIDI), BTK (CODI), and DDR1 (CIDO). The RMSD values were measured for Cα of these structural elements with respect to the holo counterparts. KiSSim distances are provided to show structural similarities to holo-structures using Minkowski’s distance on KiSSim pocket structural features. Upward arrows indicate that the AF2 structure is more similar to top performing crystal holo structure (KiSSim distance) compared to the apo one, while downward arrows indicate that the AF2 structure is more similar to the crystal apo structure. Δ LogAUC indicates the difference of LogAUC between the holo structure and the AF2 or apo structure.

We observe that the best enrichment is found for ABL1, predicted in the CIDI state (Fig. 4A). Importantly, the AF2 model performs slightly better than the best holo structure in the early enrichment of known actives (LogAUC: 252.54 versus 249.44). This is surprising considering that all AF2 models are apo structures. On the other hand, the worst match is given by DDR1, predicted in the CIDO state (Fig. 4C). CIDI structures are the most represented conformational state present in the PDB (Fig. 1), while CIDO are the second last in terms of representation. From a visual inspection of the models, we observe that AF2 reproduces the state-specific arrangement of binding site residues needed to recognize active molecules for the CIDI conformation, while the poorest models can be observed for the CIDO conformation (Fig. 4D–F).

**Figure 5.**
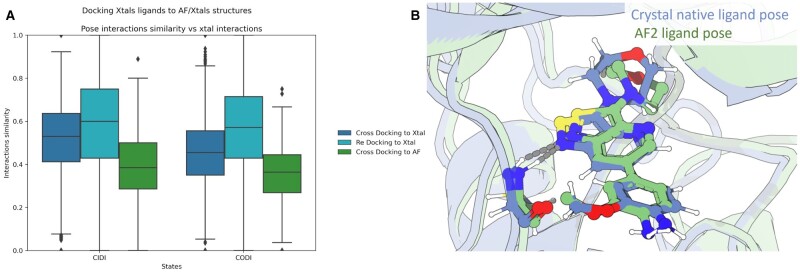
(A) Evaluation of kinase–ligand binding site interactions of AF2 structures. Ligands co-crystallized with kinases in the CIDI and CODI conformations where re-docked into their original structure, or cross docked into a conformation-matched crystal structure or the respective AF2 model. Interaction similarity is defined as the Jaccard coefficient between the interactions observed in the docked structures to the interactions observed in the native structure. (B) Pose of highest performing AF2 model in terms of interaction accuracy versus native ligand crystal pose.

Finally, we evaluated the performance of AF2 models in maintaining known kinase–ligand interactions in the binding pocket (Fig. 5). Compared to both redocking and cross-docking experiments of crystal ligands to conformation-matched structures of kinases, AF2 models consistently perform the poorest in recapitulating all known kinase–ligand interactions.

Taken together, in case of high representation of a conformational state in the PDB, the AF2 structures provide comparable early enrichment in the identification of true actives in the docking benchmark set compared to the minimized crystal structures of the same conformational state (Fig. 4). On the other hand, AF2 structures may lose key interactions that facilitate ligand binding, which may directly impact the prioritization molecules during activities, such as virtual screening (Fig. 5). Critically, the amount of representation of a given conformational state in the PDB correlates with enrichment performance, and thus the quality of the generated structure.

## 4 Discussion

Elucidating the conformational landscape of protein kinases may lead to key insights into cellular signaling mechanisms as well as enable the discovery of more effective therapeutics. We investigated the capability of AF2 to model protein kinases across several conformational states. Given that the majority of kinase structures in the PDB are in the active conformation (Fig. 1A), we initially hypothesized that AF2 may confidently model just the active conformation of kinases. Surprisingly, AF2 is able to model several conformational states of protein kinases, and certain conformations are observed in specific kinase families (Fig. 1B). This observation is due to CIDO, CODI, CODO, and ωCD conformations often being stabilized with a ligand bound (Roskoski 2016). Thus, kinase families enriched with known drug targets are privileged in terms of conformational diversity in both native crystal and AF2-modeled structures. Importantly, the AF2 models maintained key evolutionary and structural relationships between kinases (Fig. 1C).

We then investigated the confidence of AF2 predictions in the 3D positioning of certain motifs that determine specific kinase conformational states. We observed that there is significant variability in pLDDT at these motifs (Fig. 2A and B). For kinases that have multiple solved crystal structures across several conformations or kinases with structures in rare conformational states, pLDDT is lower on average at these motifs than to the entire kinase domain (Fig. 2C and D). We propose that the variability of pLDDT in AF2 models may, in part, be explained by the conformational diversity of individual kinases appearing during the training process. The relationship between conformational flexibility and pLDDT is supported by an anti-correlation between B-factors and pLDDT values in ABL1 crystal structures (Supplementary Fig. S1). MD simulations additionally reveal that atomic fluctuations are also anti-correlated with pLDDT (Fig. 3 and Supplementary Fig. S2), except for the DFG motif, which appears to be rigid and requires longer simulations or enhanced sampling (Vani *et al.* 2023) to capture its switching behavior.

Finally, we performed docking against both AF2 and conformation-matched protein kinase crystal structures to evaluate the utility of AF2 models in virtual screening. We observe that analyzing the conformational states of AF2 kinase structures separately is critical for proper benchmarking of these models (Fig. 4). In early enrichment of known ligands, the CIDI AF2 models, specifically the AF2 model of ABL1 kinase, performed comparably to the best holo crystal structure. Furthermore, we observed that the binding site residues of this predicted structure were accurately modeled by AF2 to match a holo active structure. On the other hand, we note drops in enrichment performance for AF2 models of inactive conformations, specifically in the CIDO structure of DDR1. We posit that this is due to the lack of CIDO representation during the AF2 training process, thus having an impact on the quality of the generated structure. While absolute ligand enrichment performance may be influenced by the modeled conformation, it is also important to note that AF2 models consistently lose protein–ligand interactions that may be involved in binding (Fig. 5). Recently, several studies have evaluated the usefulness of AF2 models for SBDD. Studies by Zhang *et al.* (2022) and Diaz-Rovira *et al.* (2023) have shown that certain AF2 models need structural refinement before being used in virtual screening campaigns. Thus, proper model preparation may be necessary to best utilize AF2 kinase constructs for SBDD.

We conclude that AF2-modeled kinase structures can be used to effectively model the conformational landscape of the kinase active site for highly represented conformational states. Importantly, recent work by Sala *et al.* demonstrates the potential to bias AF2 to generate models of kinases in specific conformational states (Sala *et al.* 2023). Taken together, we see a large opportunity to leverage AF2 to rationally design novel, conformation-specific inhibitors for kinases lacking solved structures.

## Supplementary Material

vbad129_Supplementary_Data

## Data Availability

The data underlying this article are available in the article and in its online supplementary material. Code used to generate figures can be found at https://github.com/Harmonic-Discovery/AF2-kinase-conformational-landscape.
