# Supplementary material for: Investigating the conformational landscape of AlphaFold2-predicted protein kinase structures
Source: Bioinform Adv. 2023 Sep 15;3(1):vbad129. doi: 10.1093/bioadv/vbad129 (PMC10541651; doi:10.1093/bioadv/vbad129)
Supplement: vbad129_Supplementary_Data [file vbad129_supplementary_data.pdf]

Investigating the conformational landscape of AlphaFold2-predicted protein kinase structures

Supplementary Data

Carmen Al-Masri[1,2], Francesco Trozzi[1], Shu-Hang Lin[1,4], Oanh Tran[1,3], Navriti Sahni[1], Marcel Patek[1], Anna Cichonska[1], Balaguru Ravikumar[1], Rayees Rahman[1,*]

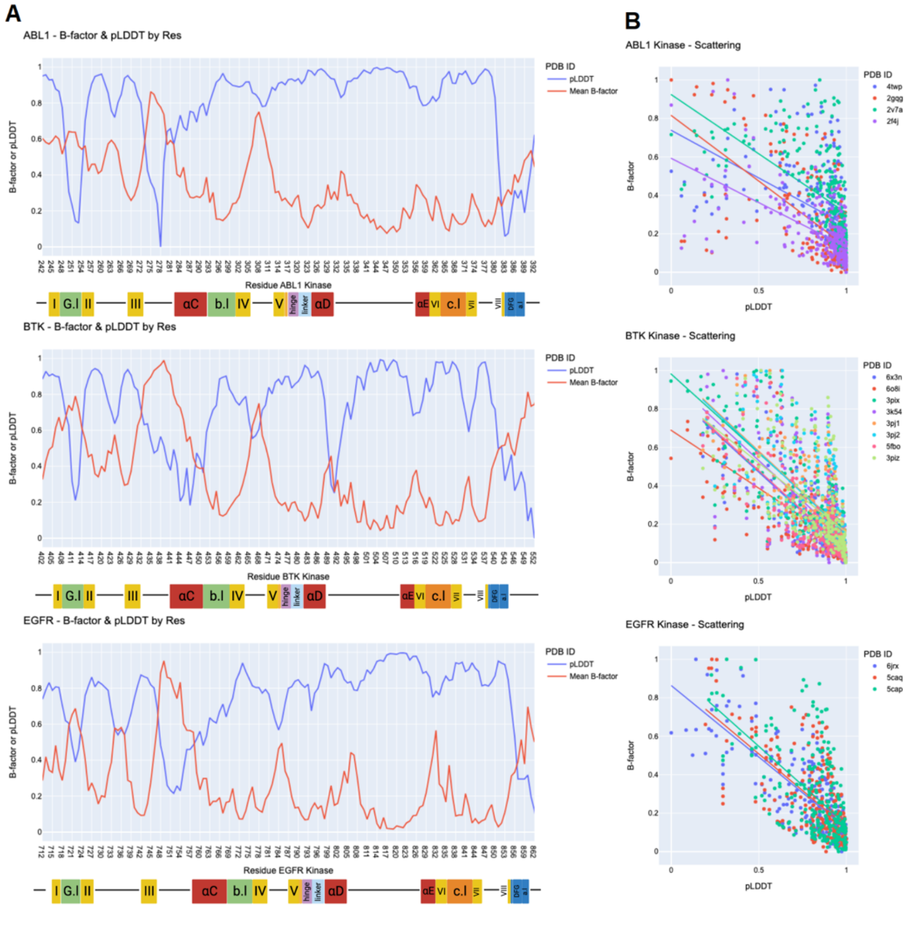

SI Figure 1: Inverse relationship between pLDDT and B-factor values in ABL1, BTK, and EGFR kinases. (A) Plots of pLDDT scores and mean B-factors against the residue numbers of the kinase. Blue line represents the pLDDT scores from AF2 model, where higher scores reflect

greater confidence. Red line represents the mean B-factors derived from CIDI PDB structures with high resolution (≤2.5Å), where lower scores reflect lower atomic mobility. An inverse relationship is observed for all three kinases, suggesting that higher AF2 confidence corresponds to lower atomic mobility, and vice versa. (B) Scatter plots of B-factor versus pLDDT for each residue with trendlines for each high-resolution PDB structure demonstrates a general inverse correlation between B-factor and pLDDT.

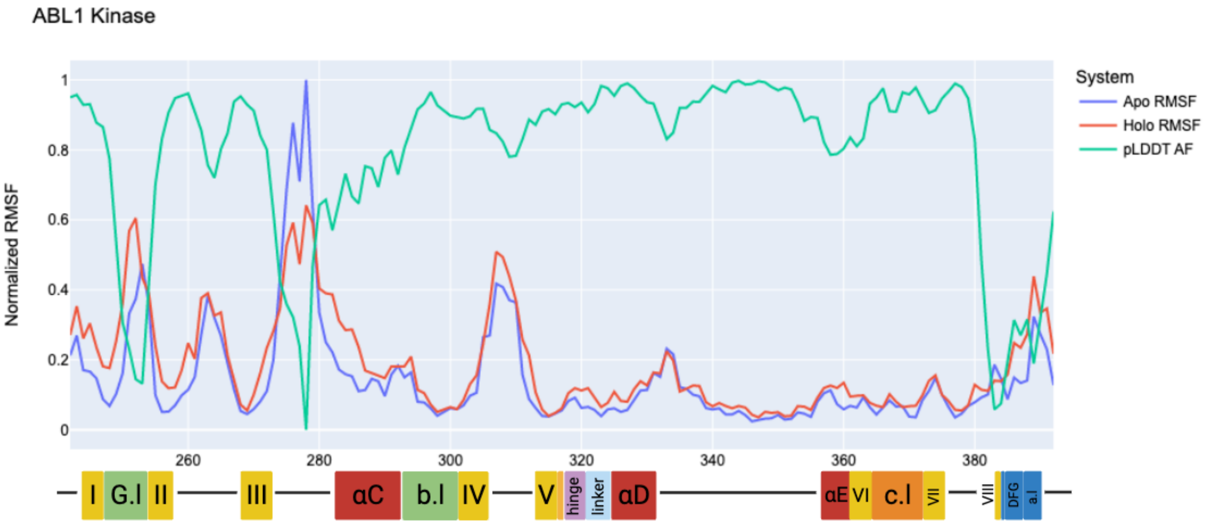

SI Figure 2: Inverse relationship between pLDDT and RMSF values from MD simulations in ABL1.

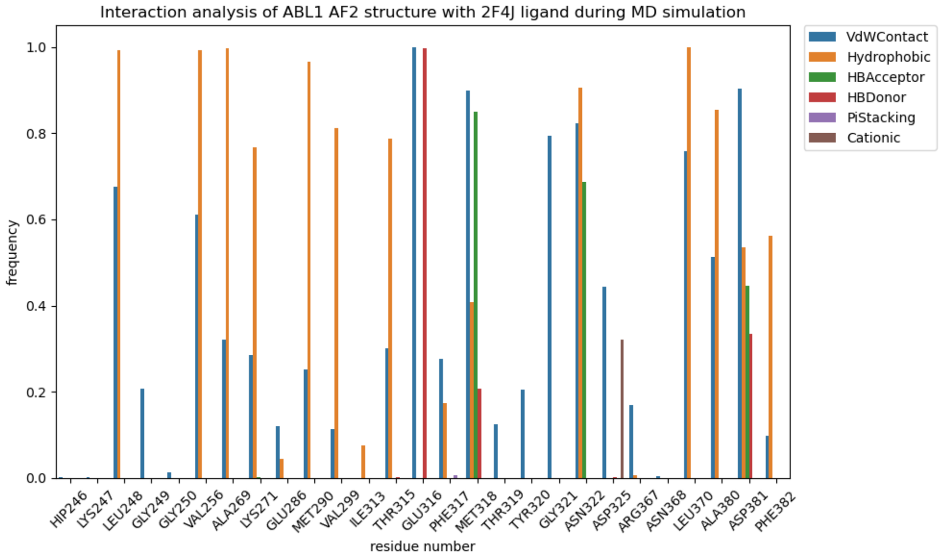

\

SI Figure 3: Frequency of interactions between ABL1 AF2 and 2F4J ligand during MD simulation. The bar plot displays the frequency (ranging from 0.0 to 1.0) of different types of

interactions occurring between the AlphaFold2-generated ABL1 kinase and the 2F4J ligand. Each bar corresponds to a specific residue within the kinase pocket.

SI Table 1: Summary of ABL1, BTK, and EGFR kinases and their respective PDB structures. The table lists each PDB structure and the resolution of each structure, along with the slope of the trendlines seen in SI Figure 1B, and the R-squared and Spearman correlation coefficients between the B-factor and pLDDT scores. These data provide a quantitative comparison of the relationship between AF2 confidence (pLDDT) and atomic mobility (B-factor) across different kinases and protein structures.

Kinase	PDB ID	Resolution (Å)	Slope	R^2	Spearman Coefficient
ABL1	4twp	2.4	-0.4952	0.208	-0.739
	2gqg	2.4	-0.6820	0.391	-0.615
	2v7a	2.5	-0.6156	0.303	-0.755
	2f4j	1.9	-0.4606	0.360	-0.541
BTK	6x3n	1.95	-0.8307	0.544	-0.663
	6o8i	1.42	-0.6017	0.458	-0.757
	3pix	1.85	-0.8213	0.577	-0.733
	3k54	1.94	-0.8429	0.424	-0.759
	3pj1	2.00	-0.8543	0.479	-0.716
	3pj2	1.75	-0.8540	0.415	-0.739
	5fbo	1.89	-0.8338	0. 557	-0.583
	3piz	2.21	-0.7225	0.312	-0.585
EGFR	6jrx	2.2	-0.7434	0.596	-0.748
	5caq	2.5	-0.7634	0.414	-0.718
	5cap	2.4	-0.8141	0.365	-0.759

SI Table 2: KiSSim distance matrix comparing ABL1's AlphaFold structure with its PDB Holo and Apo structures

	ABL1 AF2	ABL1 2F4J (Holo)	ABL1 6XR6 (Apo)
ABL1 AF2	0	42.37	62.25
ABL1 2F4J (Holo)	42.37	0	52.15
ABL1 6XR6 (Apo)	62.25	52.15	0

SI Table 3: KiSSim distance matrix comparing BTK's AlphaFold structure with its PDB Holo and Apo structures

	BTK AF2	BTK 7L5P (Holo)	BTK 1K2P (Apo)
BTK AF2	0	75.22	48.81
BTK 7L5P (Holo)	75.22	0	82.82

BTK 1K2P (Apo)	48.81	82.82	0

SI Table 4: KiSSim distance matrix comparing DDR1's AlphaFold structure with its PDB Holo structure

	DDR1AF2	DDR1 6FEX (Holo)
DDR1 AF2	0	33.03
DDR1 6FEX (Holo)	33.03	0

Tables 2-4 present the KiSSim distance matrices for the kinases ABL1, BTK, and DDR1, each comparing its respective AlphaFold model to its PDB structures. The KiSSim distance metric quantifies the structural dissimilarity between different protein structures, taking into account their physicochemical and spatial properties. Lower KiSSim distance values indicate greater structural similarity.
